# Structural basis of inactivation of human counterpart of mouse motor neuron degeneration 2 mutant in serine protease HtrA2

**DOI:** 10.1042/BSR20181072

**Published:** 2018-10-05

**Authors:** Ajay R. Wagh, Kakoli Bose

**Affiliations:** 1 Advanced Centre for Treatment, Research and Education in Cancer (ACTREC), Tata Memorial Centre, Kharghar, Navi Mumbai 410210, India; 2 Homi Bhabha National Institute, Training School Complex, Anushaktinagar, Mumbai 400094, India

**Keywords:** hHtrA2S276C, mnd2, serine proteases

## Abstract

Serine protease high temperature requirement protease A2 (HtrA2) is involved in apoptosis and protein quality control. However, one of its murine inactive mutants (S276C aka mnd2) is associated with motor neuron degeneration 2. Similarly, this conserved mutation in human HtrA2 (hHtrA2) also renders the protease inactive, implicating pathogenicity. However, the structural determinants for its inactivation have not yet been elucidated. Here, using multidisciplinary approach, we studied the structural basis of inactivity associated with this mutation in hHtrA2. Characterization of secondary and tertiary structural properties, protein stability, oligomeric properties, and enzyme activity for both wild-type and mutant has been performed using biophysical and functional enzymology studies. The structural comparison at atomic resolution has been carried out using X-ray crystallography. While enzyme kinetics showed inactivity, spectroscopic probes did not identify any significant secondary structural changes in the mutant. X-ray crystallographic analysis of the mutant protein at 2 Å resolution highlighted the significance of a water molecule that plays important role in mediating intermolecular interactions for maintaining the functional ensemble of the protease. Overall, the crystallographic data along with biophysical and enzymology studies helped decipher the structural basis of inactivity of hHtrA2S276C, which might pave way toward further investigating its correlation with aberration of normal cellular functions, hence pathogenicity.

## Introduction

HtrA2 belongs to the high temperature requirement factor A (HtrA) family of serine proteases, which are conserved from prokaryotes to humans. HtrA was originally identified as a heat-shock-induced serine protease in *Escherichia coli* that degraded misfolded/unfolded proteins formed during excessive stress conditions [1]. The members of the HtrA family share common domain arrangement such as chymotrypsin-like serine protease domain and at least one or two C-terminal PDZ [postsynaptic density protein (PSD95), *Drosophila* disc large tumor suppressor (Dlg1), and zonula occludens-1 protein (zo-1)] or protein–protein interaction domain(s) [2]. Once these regulatory PDZ domains recognize misfolded/damaged proteins or any other interacting partner with a specific function, a series of conformational changes with concomitant activation of the protease take place [3].

The members of HtrA family are found to be associated with several critical biological functions, as well as pathogenicity, such as protein quality control, unfolded protein response, cell growth, apoptosis, and disorders including Alzheimer’s, Parkinson’s, arthritis and cancer [3–5]. Human HtrA2, the most well-known among the four human HtrA family members (HtrA1–4), has been found to exhibit proapoptotic activity with the ability to stimulate apoptosis through multiple pathways [3]. While hHtrA2 predominantly resides in the intermembrane space of the mitochondria, it has also been reported to be present in the endoplasmic reticulum (ER) and Golgi bodies [6]. Human HtrA2 is synthesized as a 458 amino acid precursor protein, comprising a short N-terminal region, a serine protease, and a C-terminal PDZ domain. The protease undergoes maturation upon removal of first 133 residues that encompass a transmembrane (TM) region and a mitochondrial localization signal (MLS). This cleavage exposes an inhibitor of apoptosis protein (IAP) recognizing tetra-peptide motif (AVPS), which is unique to this member of the HtrA family. Under apoptotic stimulation, a mature processed form of hHtrA2 (Δ133) is released into the cytosol where it binds to the cytosolic IAPs and negates their caspase-inhibitory activity [7] by direct proteolytic cleavage [5]. The mature protein has also been found to stimulate apoptosis-like morphological changes and extensive cell death that are not blocked by caspase inhibitors [5], suggesting it can induce cell death through multiple pathways.

Structurally, mature hHtrA2 exists as a homotrimer with pyramidal architecture where the core serine protease domains are surrounded by C-terminal PDZ domains that reside at the base. The short N-terminal regions that form the top of the pyramid involve in packing of the three monomers primarily through van der Waals interactions thus creating an extremely buried active-site milieu. HtrA2 consists of 7 α-helices and 19 β-strands, which together form well-defined domains along with several functionally important loop structures [8]. Among these loops, the active site serine residue (S306) resides in Loop 1 (L1; 302-306), while Loop 3 (L3; 275-285) harbors the mnd2 mutation. Apart from rendering flexibility to the trimeric protease ensemble, these loops that together form the activation domain have also been implicated in allosteric modulation of serine protease activity of hHtrA2 [3,9]. A close look at its bacterial counterpart, DegP, shows that it exhibits major rearrangement upon ligand binding in the sensor loop L3. This results in its interaction with the activation loop LD of a neighboring subunit (referred to here as LD*), thereby inducing disorder-to-order transition of the activation domain [3]. The structural signature as well as the dynamics associated with this mechanism of activation is conserved in the HtrA family including hHtrA2 that requires a coordinated action of the regulatory loops. The PDZ domain in hHtrA2 is connected to the protease domain via a flexible linker sequence and has a peptide binding groove (YIGV, a variation of GLGF motif) formed by β14 and α7 structures. The peptide-binding pocket of the PDZ domain is buried at the interface between the PDZ and the protease domains suggesting requirement of a significant conformational change for binding of the C-terminal interacting partners.

Recently, increasing evidences have linked hHtrA2 to neurodegeneration and the cellular protein quality control system. Motor neuron degeneration 2 (mnd2) homozygous mice, in which the missense mutation S276C leads to a remarkable loss of murine HtrA2’s protease activity, showed severe neurodegeneration in the striatum and less severe neurodegeneration in the brain stem and spinal cord [10]. Interestingly, several missense mutations, in the gene coding for hHtrA2, were reported to be associated with Parkinson’s disease (PD) [11]. In consonance with these reports, hHtrA2 has also been found in neurons and glial cells in brains with α-synucleinopathies [12] as well as in Lewy bodies [11]. Similar to mnd2 mice, hHtrA2 knockout mice showed loss of a population of neurons in the striatum, with PD phenotype leading to death within 30 days of birth [13]. Additionally, many other neurodegenerative disease proteins, such as presenilin-1 [14,15] and amyloid precursor protein, [16] have been reported to be associated with hHtrA2. Furthermore, loss of hHtrA2 activity in non-neuronal tissues has recently been shown to cause premature aging [17].

However, apart from the mouse model study, no further mechanistic characterization has been done on S276C mutant to delineate the structural basis of its inactivation. Therefore, the aim of the present work is to understand how S276C point mutation that resides away from the catalytic triad completely abolishes the protease activity, which would indirectly shed light on hHtrA2 mechanism of action. In HtrA family, it is known that the sensory loop L3 (that harbors S276), upon substrate binding, rearranges and carries out intermolecular interactions, which ultimately leads to enzyme activation [3]. Similarly, sensory loops have been found to play crucial functional roles in hHtrA2 as well [9,18]. Therefore, mutation of serine 276 to a cysteine in hHtrA2 might destabilize the intermolecular interaction networks involving loop L3 and adjoining residues and loops. The present study reports the crystal structure of hHtrA2S276C protease at 2 Å resolution with intact catalytic triad. Comparison with hHtrA2 structure suggests that the absence of a critical water-mediated intermolecular interaction between side chains of S276 of L3 and I270 from LD* (of adjoining monomer) might abrogate the relay of signal towards loop L1*, which include residues of oxyanion hole and catalytic triad thus resulting in an inactive variant of the protease.

## Materials and methods

### Sub cloning, protein expression, and purification

Recombinant mature (Δ133) hHtrA2 with C-terminal 6X-Histidine tag in pET-20b (Addgene, Cambridge, MA, U.S.A.) vector was expressed and purified as described previously [19]. To introduce mutations (S276C, S276A, R280A, and S306A), site-directed mutagenesis (SDM) (Stratagene, TX, U.S.A.) was performed. Primers used for SDM are shown in Supplementary Table S1. Mutations were confirmed by Sanger DNA sequencing.

Recombinant proteins (wild-type [wt] and hHtrA2S276C, S276A, R280A, and S306A) were expressed in *E. coli* strain Rosetta (DE3) (Novagen, Billerica, MA, U.S.A.). Cells were grown at 37°C until OD_600_ of 0.6 was reached and then induced with 0.25 mM IPTG. Cells were further cultured at 16°C for 20 h postinduction. Proteins with the 6X-His tag were purified by affinity chromatography using nickel–nitrilotriacetic acid matrix (Ni-NTA) (Novagen), in 20 mM Na_2_HPO_4_/NaH_2_PO_4_ (pH 8.0) buffer containing 100 mM NaCl (referred to as buffer-A hereafter). Mutant hHtrA2 proteins were further purified through a gel filtration column (Superdex^®^ 200 10/300 GL; GE AKTA purifier) as a part of a two-step purification. The purity of the eluted fractions was checked on 12% SDS–PAGE. During the procedure of crystallization of hHtrA2S276C and S306A, proteins were concentrated using an Amicon ultra centrifugation unit (Millipore, molecular-weight cutoff 10 kDa) to get a maximum concentration of 25 mg/ml.

### *In vitro* protease activity assays

Protease activity of mature wt hHtrA2 and its mutants (S276C, S276A, and R280A) was measured by in-gel as well as continuous fluorescence-based protease assays. The in-gel protease assay was carried out as described previously [20,21]. Protease activity of all the hHtrA2 variants was determined using substrate β-casein (Sigma Chemicals, St Louis, MO, U.S.A.) and compared with the wt hHtrA2 (used as a positive control) and catalytically inactive mutant (S306A) of hHtrA2 (used as a negative control). For each 20 µl of reaction mixture, mutant protein was incubated with 10 µg of β-casein in buffer-A at 37°C for 60 min and results were analyzed by SDS–PAGE. For all quantitative studies, FITC β-casein (Sigma) was used as described earlier [20]. Reaction rates (*V*_o_) at different substrate concentrations were calculated using linear regression analysis. Steady state kinetic parameters were determined by fitting the data to the Hill form of the Michaelis–Menten equation:

$$\mathrm{Velocity}\;(V)=\frac{V_{\max}}{\left[1+\left(\frac{K_{0.5}}{\left[\mathrm{substrate}\right]}\right)n\right]}$$

where *V*_max_ is the maximum velocity, ‘*n’* is the Hill constant, and *K*_0.5_ is the substrate concentration at half maximal velocity using KaleidaGraph (Synergy Software, Reading, PA, U.S.A.) as previously mentioned [9,20].

### Native-PAGE assay

Purified proteins (wt and hHtrA2S276C) in native-PAGE sample buffer (62.5 mM Tris-Cl, pH 6.8, 15% glycerol, and 0.01% bromophenol blue) were separated on a 7.5% native-PAGE. Samples were run along with the standard protein ladder of molecular weight (MW) (NativeMark™ Unstained Protein Standard, Thermo Fisher Scientific, Inc.) ranging from 40 to 150 kDa.

### Gel filtration chromatography

Gel filtration chromatography was performed in a buffer comprising 10 mM HEPES (pH 8.0), 100 mM NaCl referred to as buffer-B hereafter. Highly concentrated (∼20 mg/ml) purified mutant proteins (S276C and S306A) were separately applied to a Superdex S200 HR 10/300 column (GE Healthcare, Uppsala, Sweden) that was pre-equilibrated in buffer-B. Proteins were then eluted in the same buffer at a flow rate of 0.2 ml/min. The standards used for calibration were as follows: BSA 66 kDa, alcohol dehydrogenase 150 kDa, and *β*-amylase 200 kDa. Elution volume (*V*_e_)/void volume (*V*_0_) compared with log of molecular masses of standards was plotted to generate the calibration curve from which MWs of hHtrA2 and its variants were calculated as described earlier [20].

### Dynamic light scattering

The dynamic light scattering (DLS) measurements were performed using the DynaPro-MS800 instrument (Protein Solutions Inc., VA, U.S.A.). The mutant hHtrA2S276C protein and buffer solutions were filtered (0.22 µm pore size) and degassed prior to measurement. Protein (3 mg/ml) in buffer-A was loaded into a 45 μl quartz cuvette. Experiments were performed at 25°C and at least 20–30 measurements each of a 10-s duration were collected. The refractive index and viscosity values were taken for water as provided by the software. Histogram analyses of DLS results were carried out using the software DYNAMICS version 6.0. The molecular size estimation in the present work was done using Wyatt technology DynaPro particle size analyzer. From the correlation function, the diffusion coefficients (*D*_T_) of the molecules were calculated by fitting the data. Finally the hydrodynamic radius (*R*_h_) of the particles and molecules were determined:

(1) $$D=\frac{kT}{6\pi \eta_{0}R_{h}}$$

(2) $$R_{h}=\frac{kT}{6\pi \eta_{0}R_{h}}$$

Here, *k* is the Boltzmann constant, *T* is temperature in Kelvin, and *η*_0_ is viscosity of solvent.

### Far-UV circular dichroism spectroscopy

Far-UV circular dichroism (CD) measurements were made using a JASCO J-815 spectropolarimeter (JASCO, Easton, MD, U.S.A.) with a 1 mm cell at 25°C in a thermostatted cell holder at a concentration of 10 μM in buffer-A. CD spectra of wt hHtrA2 and purified hHtrA2S276C were recorded in the far-UV region (260–195 nm). All spectra were recorded at least thrice and the average is plotted. The mean residue ellipticity [*θ*] mrw, *λ* (deg·cm^2^·dmol^−1^) is given by

(3) $$[\theta ]\mathrm{mrw},\;\lambda =\frac{\mathrm{MRW}\theta }{10d.c}$$

where *θ* is the observed ellipticity (degrees), *d* is the path length (cm), *λ* is the wavelength (nm), and *c* is the concentration (g/ml).For thermal denaturation studies, far-UV CD experiments were performed between 25 and 100°C at 2°C intervals. Thermal denaturation studies were performed to measure melting point temperatures (*T*_m_) of wt and hHtrA2S276C. Far-UV CD signals were recorded between 20 and 85°C at 2°C intervals at 208 nm. The mean residue ellipticity was computed as reported previously [22].

### Protein crystallization

Concentrated protein samples of hHtrA2S276C and S306A (∼25 mg/ml) were used for crystallization. Initial crystallization trials were performed by hanging-drop vapor-diffusion method in 48-well crystallization plates (Hampton Research, Laguna Niguel, California, U.S.A.) utilizing the commercial crystallization screens: Screen 1 and Screen 2 (Hampton Research). Drops prepared by mixing 2 µl of protein solution with an equal volume of crystallization buffer were equilibrated against 500 µl of crystallization buffers.

Initially, crystal-like particles were observed in one of the wells containing formulation number 21 (0.1 M MES pH 6.5 (buffer), 2 M NaCl (Precipitant), 0.1 M KH_2_PO_4_, and 0.1 M NaH_2_PO_4_) from Crystal Screen 2 (Hampton research) postincubation at 295 K for 2 weeks. For the crystallization of hHtrA2S306A, the same condition was followed. Further finer optimization of crystallization conditions led to the formation of larger crystals. Finally, equilibration of a mixture of 2 µl of protein solution with 1 µl of precipitant solution against reservoir solution at 295 K for 15 days produced diffraction-quality crystals, with a biggest dimension of 0.3 × 0.2 × 0.2 mm. The crystals were cryo-protected using ultrapure glycerol (Hampton Research).

### X-ray diffraction data collection and processing

The single-crystal diffraction experiments on hHtrA2S276C mutant protein crystals were carried out on the protein crystallography beam line (PX-BL21) at the 2.5 GeV Indus-2 synchrotron radiation facility RRCAT, Indore, India [23]. The crystals were soaked in a cryo-protectant solution (reservoir/well solution with 30% glycerol). A total of 131 diffraction images for S276C crystals were collected on a MAR225 CCD (Rayonix) detector from a cryo-cooled crystal (100 K) by using 1° oscillation and X-rays of wavelength 0.97947 Å.

For hHtrA2S306A, multiwavelength X-ray data were collected at the beamline BM30 (FIP), ESRF, and Grenoble. All data sets were collected using the single, flash-frozen crystal of hHtrA2 S306A of size 0.35 × 0.45 × 0.2 mm. Data were indexed and integrated using software **X**-ray **D**etector **S**oftware (XDS) and were subsequently scaled using the software AIMLESS from the CCP4 suite [24].

### MD simulation and analysis

The coordinates of the initial structure used in the present study are from the crystal structure of hHtrA2S306A (1LCY) [8] obtained from the protein data bank (PDB) [25]. The crystal structure (1LCY) has missing N-terminal residues (AVPSP) and two flexible regions (^344^RGEKKNSSSGISGSQ^358^ and ^282^ARDLGLPQT^290^); therefore, we modeled and refined the missing loop regions using Prime 3.0 (Schrodinger, LLC, New York, 2011).

Accordingly, we created S276C on the same template for our further MDS studies. We subjected both the proteins, wt hHtrA2 and hHtrA2S276C), to molecular dynamics simulation (MDS) run for 10 ns each using GROMACS suite [26–28]. For each structure, all water molecules within 3.0 Å of a nitrogen or an oxygen atom were included in the search. All calculations were run under periodic boundary conditions using an orthorhombic (minimum distance between protein and cell faces was initially set to 10 Å) cell geometry.

## Results

### Determining oligomeric property of hHtrA2S276C

The crystal structure of catalytically inactive mutant (Ser306Ala) of hHtrA2 (PDB: 1LCY) demonstrates that Serine 276 is located near one of the residues involved in trimerisation (Phe 256), as shown in Supplementary Figure S1 [8]. To investigate whether S276C mutation affects the trimeric structure of the protein, we determined its apparent MW using native-PAGE, size exclusion chromatography (SEC) and DLS.

**Figure 1:**
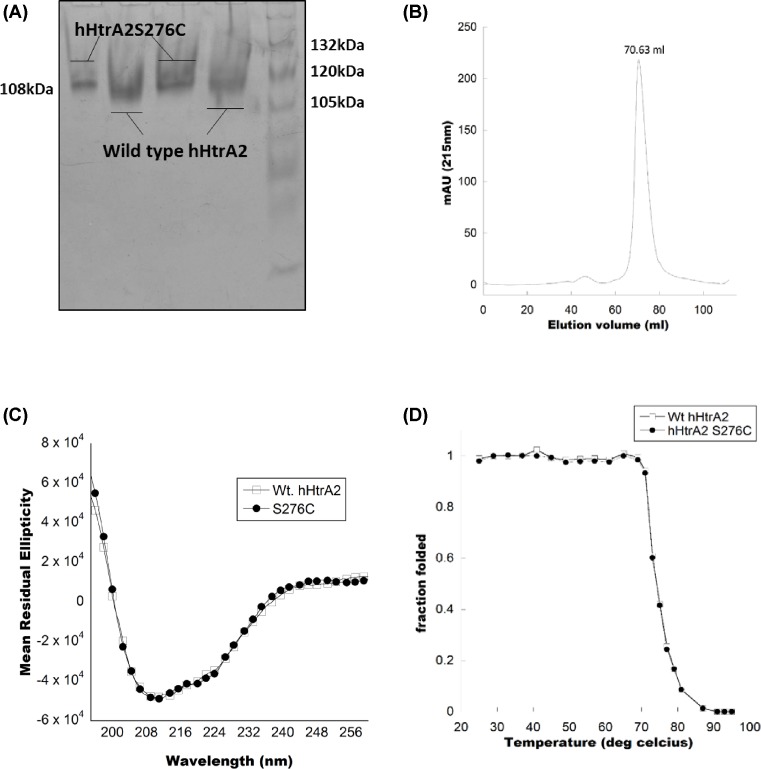
Effect of S276C mutation on the overall conformation of hHtrA2 protease (**A**) A 7.5% native-PAGE resolving gel visualized by staining with coomassie brilliant blue shows similar band size for both the proteins. (**B**) SEC showing elution peak of hHtrA2S276C at approximately 70 ml that corresponds to approximately 108 kDa as described under ‘Materials and methods’ section. (**C**) CD analysis of hHtrA2S276C protein. The far-UV CD spectra of wt hHtrA2 and hHtrA2S276C between 195 and 260 nm show very similar secondary structural architecture. The plots are average of data obtained from experiments done in triplicate. (**D**) Thermal denaturation experiment of wt hHtrA2 and hHtrA2S276C using far-UV CD spectroscopy within the temperature range of 20–100°C.

In the native-PAGE, comparison of mobility of both the bands (wt and mutant) with the standard markers depicted that the proteins are of equal MW (Figure 1A). However, native-PAGE separates proteins depending upon the charge they have and wt hHtrA2 bands displaced slightly down relative to wt hHtrA2. Furthermore, to validate the results of native-PAGE experiment, quantitative approaches have been used such as SEC and DLS. In SEC, both the wt and mutant hHtrA2 were eluted, and detected as single peaks at approximately 70 ml of elution volume (Figure 1B), similar oligomeric status for wt hHtrA2 has been observed previously [20], suggesting that the mutation does not affect the oligomeric property of hHtrA2 protein. Our DLS data also showed that mutation did not affect the overall conformation as the hydrodynamic radii of both wt hHtrA2 and hHtrA2S276C were found to be very similar [18]. Furthermore, DLS analysis also provided average polydispersity of 12.5% at 25°C for hHtrA2S276C indicating its homogeneity (Supplementary Figure S2).

### Secondary structural organization and thermal stability of hHtrA2S276C protein

To investigate whether S276C mutation results in alteration in the overall secondary structural organization of the protein or its stability, far-UV CD spectroscopy was performed on purified wt and hHtrA2S276C as observed in Figure 1C. The recorded spectra that are typical for proteins with α-helical and β-stranded structure [20] overlap on each other suggesting no significant secondary structural changes have occurred upon mutation. To determine whether S276C mutation has affected thermal stability of hHtrA2, both wt and the mutant were subjected to thermal denaturation conditions between 20 and 85°C to obtain the melting temperature. Change in ellipticity at 208 nm as a function of temperature has been represented in Figure 1D, which show a *T*_m_ value of hHtrA2S276C to be 74°C, which is comparable to that of wt hHtrA2 as reported earlier [20]. The present study indicates that hHtrA2 retains its stability upon S276C mutation and it caused neither destabilizing nor stabilizing effect on the overall structure of protein.

### Important L3 loop residues in hHtrA2 activity

**Figure 2:**
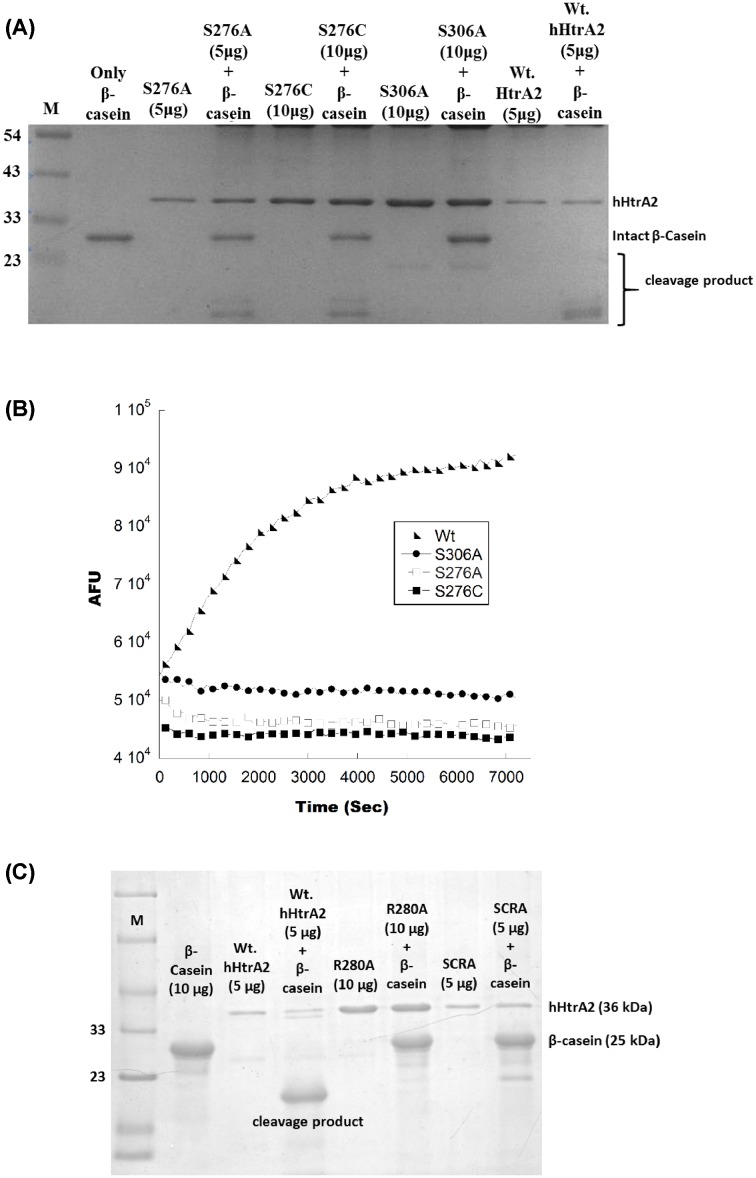
Effect of conserved L3 loop residues (S276 and R280) on protease activity (**A**) A 12% SDS gel showing *in vitro* (gel based) β-casein (substrate) cleavage assay for hHtrA2S276A. (**B**) *In vitro* (fluorescence based) protease assay for hHtrA2S276A; here hHtrA2S306A (negative) and wt hHtrA2 (positive) are used as controls. (**C**) *In vitro* (gel based) β-casein cleavage assay for hHtrA2R280A. SCRA: S276C + R280A double mutant. Loss of activity was observed in hHtrA2S276A and hHtrA2R280A mutants at given enzyme concentrations.

To understand the importance of the serine residue at position 276 toward hHtrA2 activity, SDM was performed to replace it with a smaller and hydrophobic alanine residue. *In vitro* substrate cleavage assay using β-casein (both gel-based and fluorescence-based) showed no activity in hHtrA2S276A (Figure 2A,B), suggesting that the serine on loop L3 might be important for positively regulating the activity of the protease. Since both the mutants (hHtrA2S276C and S276A) were found to be inactive, we could not calculate the reaction rate of catalysis; however, we have presented the kinetic parameters for the wt hHtrA2 as shown in Supplementary Table S2, which is comparable to previous literature reports [20].

With an aim at understanding the importance of loop L3 (on which S276 is located) for protease activity, the residues of the loop were scanned and the conserved residues were identified. Among some of the previously identified residues, Arginine 280 was found to be highly conserved across species. Crystal structure of liganded and unliganded DegS and hHtrA1 [29,30] demonstrate that upon substrate binding, this conserved arginine residue interacts with residues in LD* leading to conformational changes at and around the active site making it more conducive for substrate binding. To understand the importance of this arginine residue in hHtrA2, we mutated it to alanine in wt hHtrA2 as well as in hHtrA2S276C (double mutant). The protease activity using substrate β-casein was monitored as a function of enzyme concentration. For all the concentrations of hHtrA2R280A and hHtrA2 (R280A, S276C), no activity was observed compared with wt hHtrA2 as shown in Figure 2C, suggesting that R280 is important for hHtrA2 activity, and the structural determinants on loop L3 that modulate HtrA activity might be overall conserved between DegS, hHtrA1, and hHtrA2.

### X-ray crystallographic structure determination and refinement

Crystallization trials of hHtrA2S306A were carried out side-by-side along with hHtrA2S276C for structural comparison under similar crystallization conditions. Both the protein crystals were of good quality and diffraction data were collected to 2.05 Å resolution. The *R*_merge_ for hHtrA2S276C in the highest resolution shell was 74.8%, the mean (*I*) or sigma(*I*) and the completeness in the highest resolution shell were 2.0 and 99.6%, respectively; therefore, the data were processed to 2.05 Å resolution.

Processing of the diffraction data using XDS [31] showed that the crystal belonged to space group H3, with unit-cell parameters (in Å) *a* = *b* = 84.38, *c* = 127.84, *α* = *β* = 90°, and *γ* = 120°. Calculation of the Matthews coefficient showed the presence of one molecule (considering the MW of 36 kDa) in the asymmetric unit, with a VM (Matthews, 1968) of 2.48 Å^3^ Da^−1^ and a solvent content of 50.36%. Data were indexed and integrated using software XDS and were subsequently scaled using the software AIMLESS from the CCP4 suite [24]. Analysis of diffraction data revealed that the crystals were twinned with the twin law (h, -h-k, -l). Molecular replacement and phasing were carried out using Phenix software [32] with the coordinates of the inactive form of hHtrA2 (hHtrA2S306A) (PDB: 1LCY, Li et al., 2002) as the search model. A test set composed of 5% of the total reflections, assigned at random, was excluded from refinement to allow calculation of the free *R* factor. The final twinned *R*_work_ and *R*_free_ were found to be 15.72 and 18.82%, respectively. No density was obtained for a part of L3 loop (residues 281–291) as well as the hinge region that connects protease to PDZ domain (344–358) and therefore they have not been included in the final model. However, the S276C mutant residue is visible in the crystal structure. Supplementary Table S3 summarizes the data collection and refinement statistics for both the crystal structures.

### Structural basis of inactivation of hHtrA2S276C mutant

**Figure 3:**
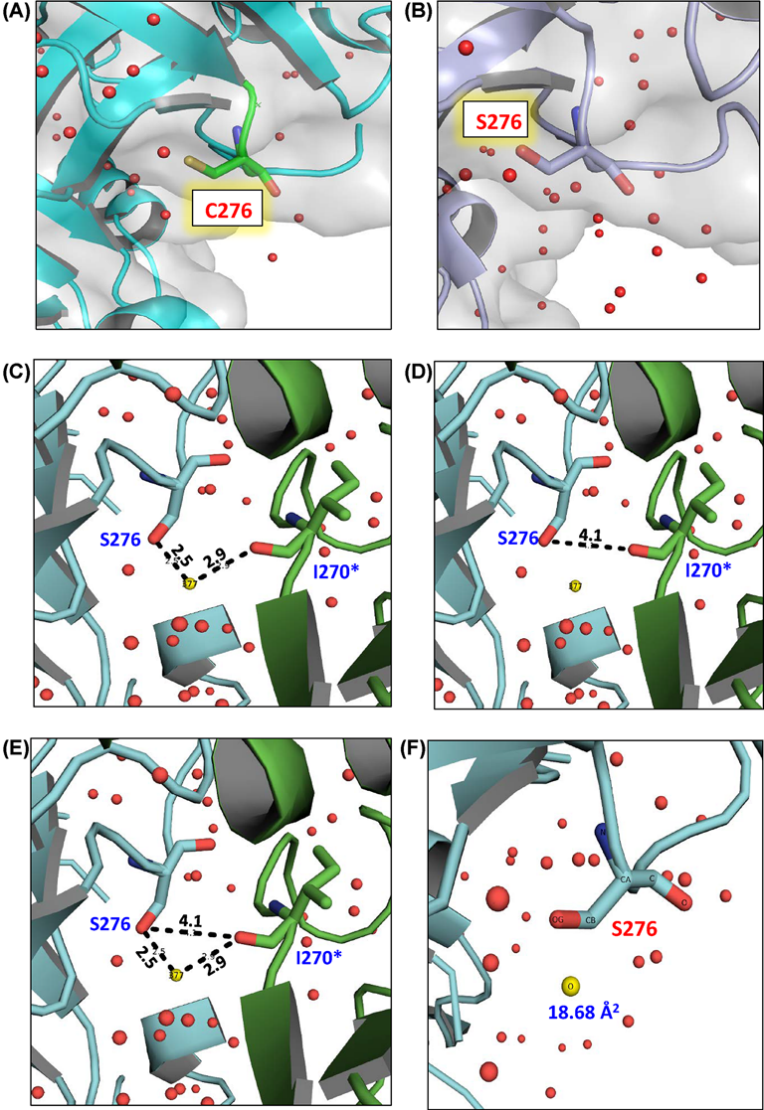
Monitoring water molecule and its interaction around cysteine 276 in hHtrA2S276C structure (**A**) hHtrA2S276C in cyan (PDB: 5WYN) depicting significantly less water molecules around C276 (indicated in green; stick model) as compared with (**B**) hHtrA2S306A (PDB: 1LCY) crystal structure (light blue). (**C**) Water molecule (W377) involve in strong H2 bonding between S276 and I270*. (**D** and **E**) Atomic distance between –OH of serine and =CO (main chain) of I270* is 4.1 Å, which in presence of W377 is forming strong hydrogen bonds. Positions of the ordered water (solvent) molecules are shown as red crosses. W377 water molecule is shown by yellow non-bonded (nb) _spheres, (**F**) W377 molecule (yellow colored) with its b-factor.

In serine proteases, the formation of the catalytic triad in an arrangement sufficiently close for electron transfer from aspartate to serine through histidine is a prerequisite for formation of an active enzyme [3]. In mature hHtrA2 (PDB: 1LCY), catalytic triad comprises His198, Asp225, and Ser306 [8]. Structural comparison of both crystal structures 1LCY and 5WYN showed that hHtrA2S276C maintains proper hydrogen bonding distances in the active-site triad residues making it compatible for catalytic activity. For example, the atomic distances between the nitrogen (ε) atom of His198 and the oxygen (γ) of Ser306 has been found to be 3.2 Å and that between the nitrogen (δ) of His198 and the oxygen (δ) of Asp228 for this structure was 2.8 Å. However, presence of C276 in L3 loop might have adversely affected the activity of the protease due to significant reduction in the number of water molecules as observed (Figure 3A) in the crystal structures of the two proteins (hHtrA2S306A PDB: 1LCY and hHtrA2S276C PDB: 5WYN) (Table 1).

**Table 1:** Comparison of number of structural water molecules of crystal structures

PDB	Resolution (Å)	Space group	Total number of water molecules	Reference
1LCY	2.01	H3	301	Li et al., 2002 [8]
5WYN	2.05	H3	190	–
–	2.01	H3	305	

A careful inspection of water molecules in the X-ray structure of hHtrA2 (PDB: 1LCY) indicates that water (W) molecule number 377 forms an interaction between side chains of S276 and I270* (distance 4.1 Å) of the adjacent molecule (Figure 3B–D). However, in case of the mutant hHtrA2S276C, where highly hydrophobic cysteine residue (CH2-SH) replaces the serine, W377 is absent suggesting abrogation of this water-mediated interaction might have adversely affected its activity. To eliminate the possibility of this observation being an artifact (as a consequence of different crystallization conditions), active site mutant hHtrA2S306A was crystallized under identical crystallization conditions as hHtrA2S276C. Superimposition of the structures of hHtrA2S306A and 1LCY (277 Cα atoms with RMSD value of 0.242 Å) confirm that the same water molecule (W3777 in case of 1LCY) is present in the hHtrA2S306A as well with a stable b-factor (temperature factor) of 18.68 Å^2^, thus highlighting its functional relevance (Figure 3E).

### Characterization of water-mediated stability using MDS

To further validate the importance of W377 molecule in stabilizing the protease structure in the dynamic loop region, MDS was performed. The coordinates of the structures used were the crystal structure of hHtrA2S306A (PDB: 1LCY) [8] and modeled structure of S276C created on the same template for further MDS analysis for 10 ns. Upon analyzing the trajectories visually and using water-mediated hydrogen bonds with important residues, we observed that in case of hHtrA2S276C, there is no water-mediated hydrogen bonding with the cysteine residue (supplementary Figure S3A and supplementary Video S1A). On the other hand, in hHtrA2S306A, there is a stable water-mediated hydrogen bonding interaction with the serine residue (supplementary Figure S3B and supplementary Video S1B), thus reiterating the importance of the water molecule (W377) in its activity.

## Discussion

The regulatory loops (mainly L3 and LD) that play critical roles in the transmission of allosteric signal and formation of activation cluster have been elaborately studied in DegS, HtrA1 and more recently in *E. coli* DegP proteases [33]. It has been found that subtle structural differences define their substrate specificity as well as their distinct mechanisms of activation, which in turn determine the explicit functions they perform within the cell. However, although the structure of hHtrA2 has been solved, intricate dissection of the loop regions with identification of critical residues involved in regulating its dynamic allosteric behavior is yet to be delineated.

Therefore, to understand the structural basis of inactivity in human counterpart (hHtrA2S276C) of mnd2 mouse mutant, high resolution crystal structure was solved followed by elucidation of its biophysical properties. Although, the mutant showed no overall secondary structural changes, conformational stability (T_m_ value ∼74°C) or conformational changes (RMSD: 0.244Å) compared with the wt, a significant reduction in the number of hydrating water molecules in the mutant was observed in the crystal structure. A close look at the structural water molecules in the X-ray structures of hHtrA2S306A (PDB: 1LCY) and hHtrA2S276C (PDB: 5WYN) show absence of a critical water molecule that mediates interaction between side chains of S276 and I270* [Water (W) molecule no. 377]. This observation was validated by solving structure of hHtrA2S306A under identical crystallization conditions as S276C mutant. It is well established that the contribution of water molecules to a protein’s three dimensional structure is phenomenal. Hydration of protein structure is very important for maintaining its overall tertiary/quaternary structure [34] as well as its biological functions [35–37]. On the contrary, disturbance in the protein–water interactions has been found to be associated with unfavorable alterations in stability or dynamics of the protein [36,37]. Depending upon the nature of the amino acids, water molecules form a network of interaction with the side chains. Side chains of hydrophobic amino acids tend to repel water molecules thereby interfering with its biological activity by abolishing the water-mediated ionic interactions. Therefore, replacement of polar serine with a more hydrophobic cysteine residue [38] might have led to shielding of the surrounding region with respect to polar water molecules thus leading to abrogation of the water-mediated interaction in hHtrA2S276C.

Human HtrA2, along with 7 α-helices and 19 anti-parallel β-strands, comprises several long loop regions (LD, LA, L1, L2, and L3) [21] (Supplementary Table S4), which play an important role in regulation of its catalytic activity [3,9,39]. Upon substrate binding, conformational changes in the sensor loop L3 in several HtrA proteins have been identified that allows them to interact with residues from LD* followed by L1* leading to a coordinated relay of information from L3 to L1* via LD*. This series of events enables the active-site to switch to a ‘proteolytically ON’ state [3,9]. Using MDS, we have earlier demonstrated that in a catalytically active hHtrA2, regulatory loops (L1, LD, and L3) shift from disordered to an ordered state [9] during the process of activation. This corroborates with the current structural data that demonstrates a water-mediated H-2 bond between S276 on L3 and I270 from LD*, which might be an important component of this allosteric pathway. Similar mechanism of water-mediated conformational transition to a functional state has been demonstrated in hemoglobin that allosterically regulates binding of oxygen [40]. Using structural biology and functional enzymology studies, we have provided insight into the role of a critical water molecule in conformational selection in hHtrA2 protease. Overall, the crystallographic data helped in deciphering the structural basis of hHtrA2S276C inactivity and its mechanism of action.

## Supporting information

**Figure 1:** 

**Figure 2:** 

**Figure 3:** 

**supplementary Video S1:** 

**supplementary Video S2:** 

## Abbreviations

CD: circular dichroism
DLS: dynamic light scattering
hHtrA2: human high temperature requirement protease A2
Htra: high temperature requirement factor A
IAP: inhibitor of apoptosis protein
MLS: mitochondrial localization signal
mnd2: motor neuron degeneration 2
PD: Parkinson’s disease
PDB: protein data bank
PDZ: postsynaptic density protein (PSD95), Drosophila disc large tumor suppressor (Dlg1), and zonula occludens-1 protein (zo-1)
SDM: site-directed mutagenesis
SEC: size exclusion chromatography
