# Supplementary figures and images for: Structural basis of inactivation of human counterpart of mouse motor neuron degeneration 2 mutant in serine protease HtrA2
Source: Biosci Rep. 2018 Oct 5;38(5):BSR20181072. doi: 10.1042/BSR20181072 (PMC6172425; doi:10.1042/BSR20181072)

Figure S1

A

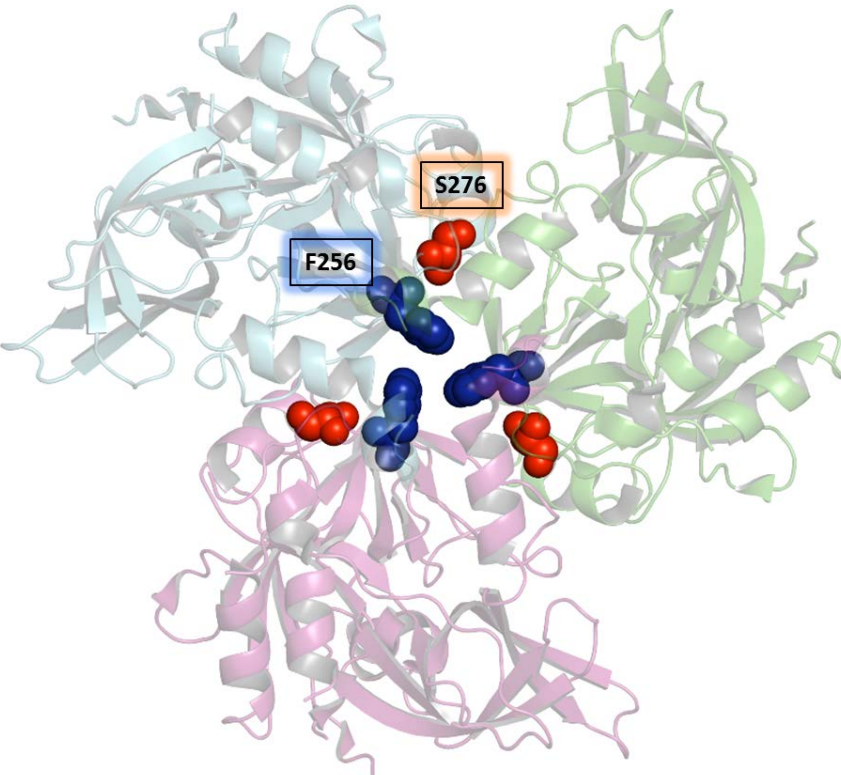

B

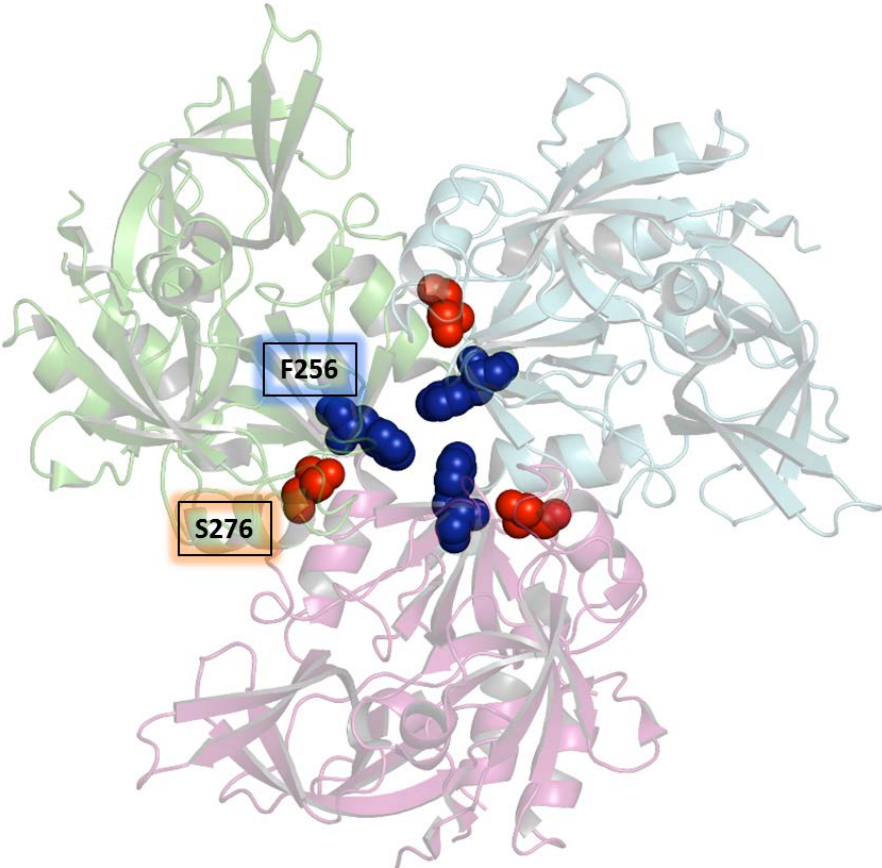

Figure S3

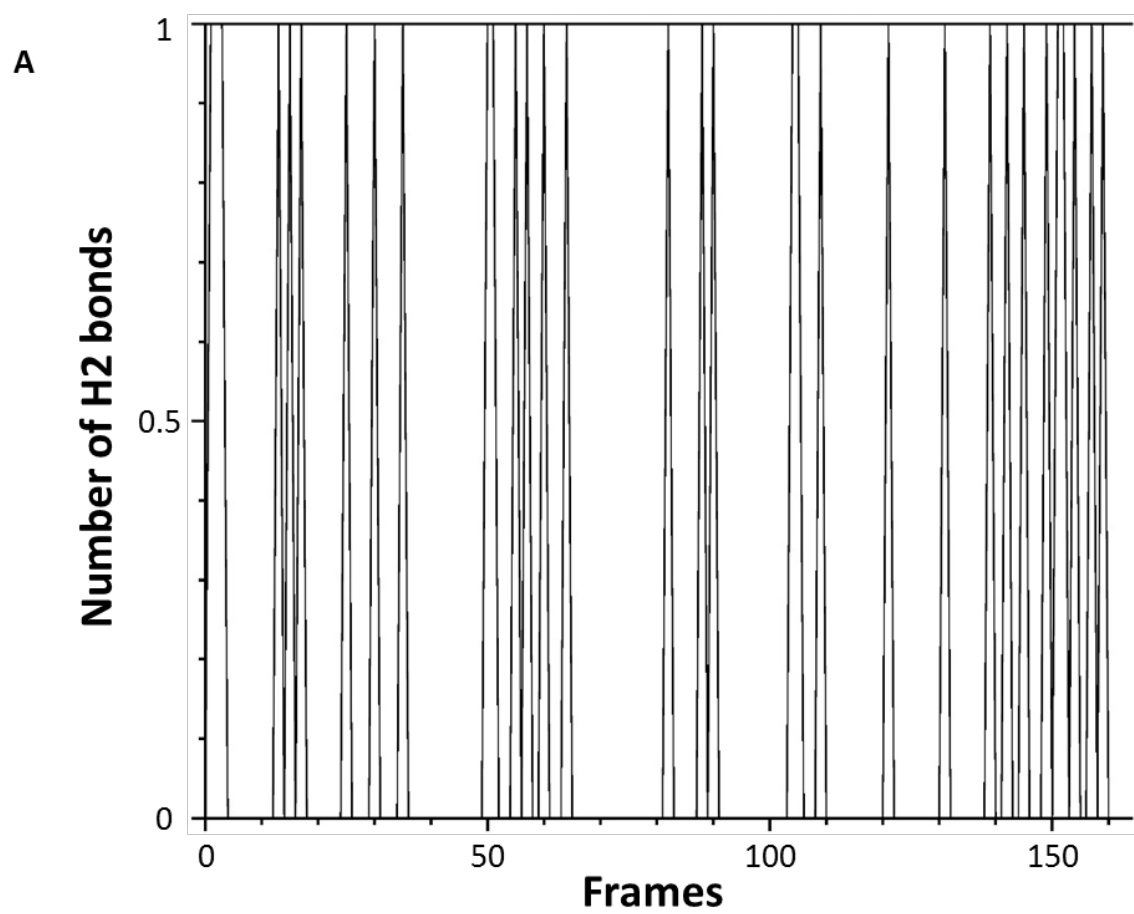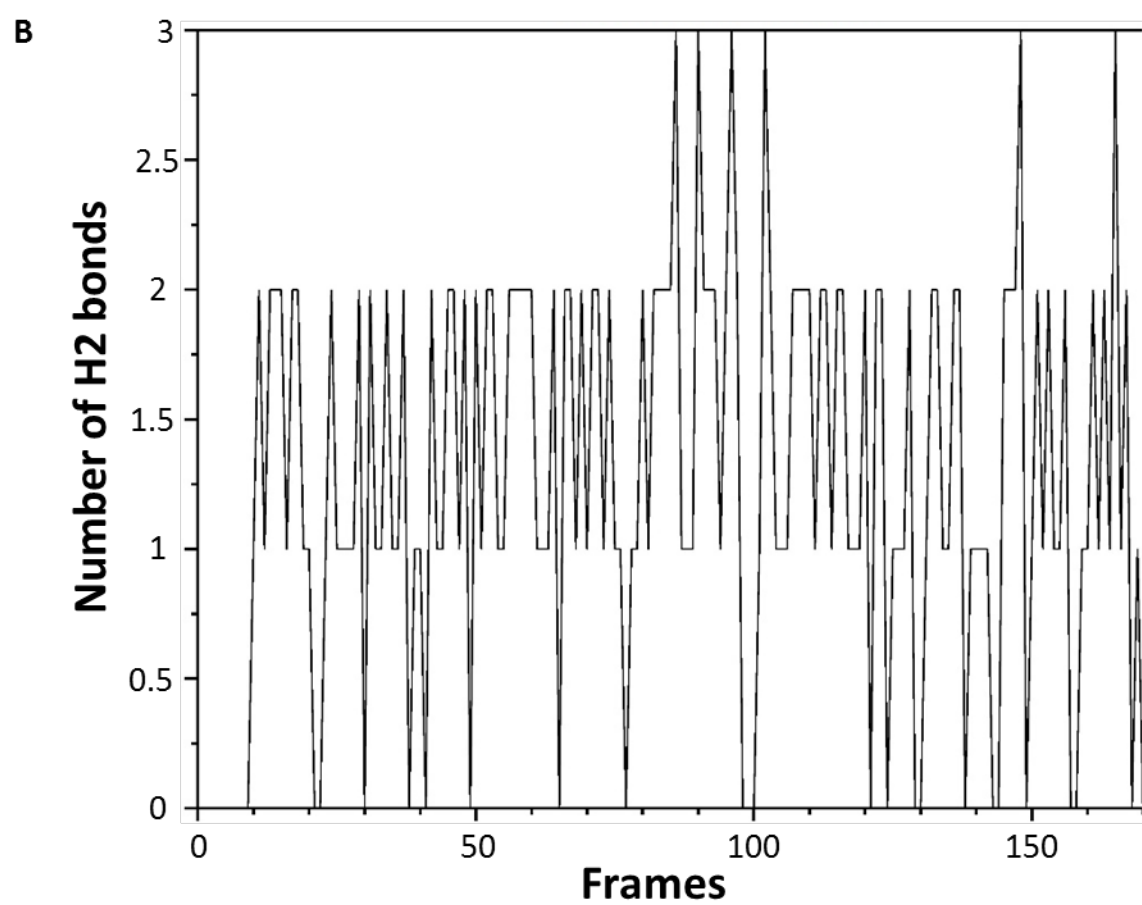

Figure S2

A

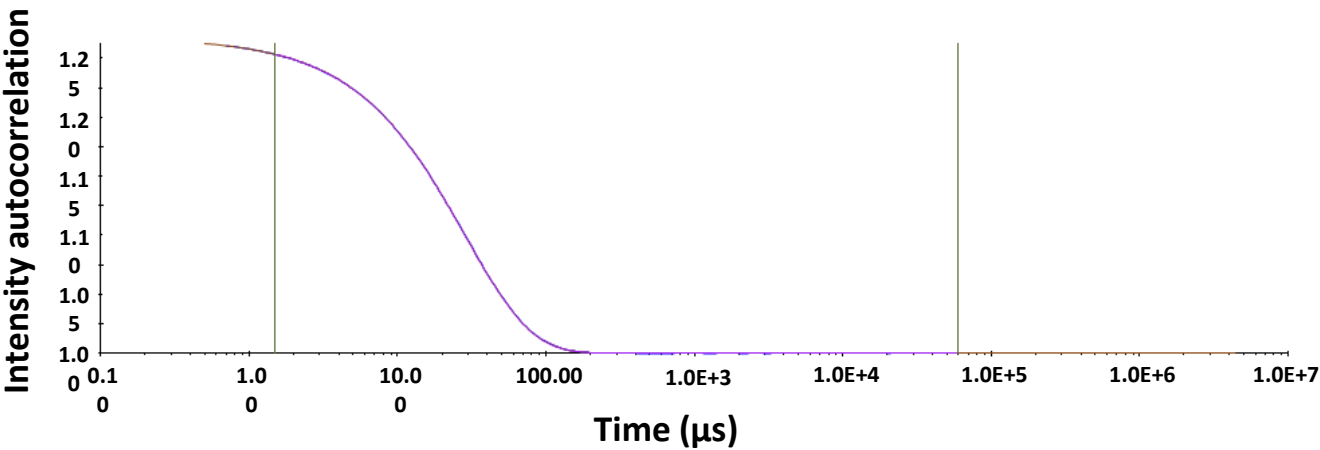

B

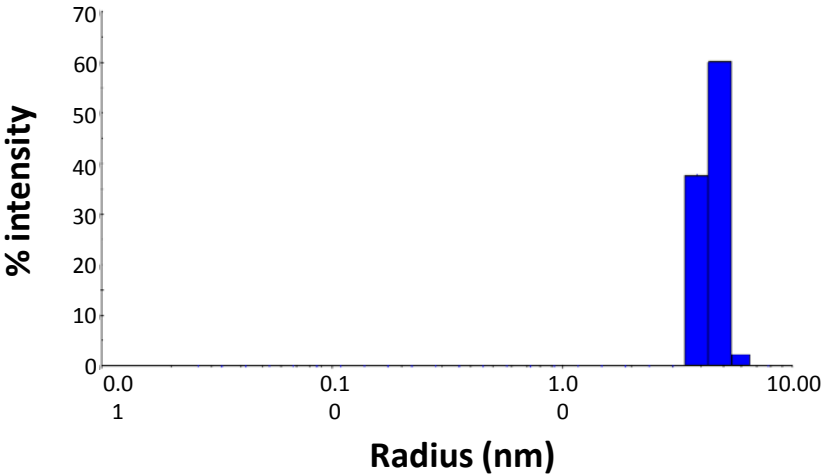

Supplement: Supplementary file 1 [file bsr20181072_Supp1.pdf]
